# A Sequence-Specific Theory for Charge-Regulating IDPs

**DOI:** 10.1021/acs.jpcb.6c00655

**Published:** 2026-04-13

**Authors:** David Beyer, Christian Holm, Zhen-Gang Wang

**Affiliations:** † Institute for Computational Physics, 9149 University of Stuttgart, D-70569 Stuttgart, Germany; ‡ Division of Chemistry and Chemical Engineering, 370767 California Institute of Technology, Pasadena, California 91125, United States

## Abstract

Intrinsically disordered proteins are a notable class of biological polymers whose physicochemical properties and biological functions are determined by an intricate interplay of chain connectivity, electrostatic interactions, the sequence of residues, and nonuniversal short-range interactions. An important phenomenon in these molecules is charge regulation, which arises from weakly acidic and basic residues, but is often neglected in theoretical descriptions. In this work, we use the Edwards–Singh variational method to derive an approximate theory that describes sequence effects in the charge regulation and chain conformation of intrinsically disordered proteins. The main result of our theory is a set of coupled algebraic equations yielding a renormalized Kuhn length and residue-specific mean-fields that determine the respective ionization states of the residues. We discuss limiting cases of these equations that underline the internal consistency of our theory and connect our results to earlier studies. To solve the full set of equations, we propose a simple numerical scheme. As test cases, we calculate the conformation and ionization state of a weak polyelectrolyte and of 30 sequence variants of the polypeptide (EK)_25_. For the weak polyelectrolyte, we show that the theory predicts phenomena such as the overall suppressed ionization due to electrostatic interactions and the enhanced ionization at the chain ends. In the case of the polypeptide (EK)_25_, we find strong effects of the sequence on ionization, with well-mixed sequences exhibiting a broad pH range where the polypeptide is net neutral, while blockier sequences exhibit a steeper ionization response. Moreover, we also observe pronounced sequence effects on the swelling behavior of the chains.

## Graphical abstract

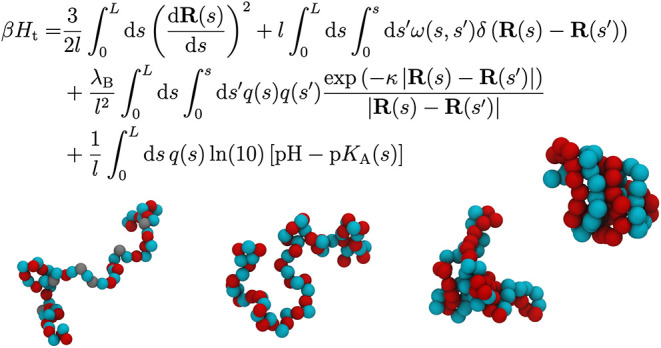

## 1 Introduction

The sequence spaces of biological polymers are astronomically large, necessitating the development and use of simplified analytical theories to understand the properties and biological functions of these macromolecules.^1–3^ Arguably, the most important class of biopolymers are proteins, i.e., polypeptide chains that are defined by a specific sequence of amino acids—the so-called primary structure.^4^ It was long considered a dogma that all proteins also exhibit higher-order structures, which means that they spontaneously fold into well-defined three-dimensional shapes. However, in recent years, it has become increasingly clear that there is a large group of proteins that lack higher-order structures.^5–7^ These “intrinsically disordered proteins” (IDPs) often contain only a small number of hydrophobic residues, but are enriched in electrically charged amino acids,^8^ and thus share many properties with synthetic polyelectrolytes and polyampholytes. Nevertheless, developing theoretical approaches for IDPs with a specific sequence is more challenging than for synthetic polyampholytes, where one almost always deals with an ensemble of sequences.^9–13^ Recently, considerable effort has been devoted to developing sequence-specific theories of IDPs that address various aspects, such as chain conformations^2,3,13–17^ and phase behavior.^18–23^

Ghosh and co-workers introduced an elegant theoretical model to describe the sequence-specific conformational behavior of IDPs.^2,3,15–17^ They used the Edwards–Singh variational method^24,25^ to derive an algebraic equation for a renormalized Kuhn length, which can be solved numerically to yield an estimate for the sequence-specific size of an IDP. The original Sawle-Ghosh theory assumed fixed integer charges on the IDP, i.e., all residues were treated as either neutral or fully ionized. However, in reality, the charged residues of a protein contain weak acid or base groups, whose ionization state depends on the pH-value and the local electrostatic environment, giving rise to the “charge regulation” mechanism.^26–35^ Theoretically accounting for charge regulation in IDPs is challenging, because the chain conformation and the ionization states of the residues are inherently coupled, resulting in a complex interplay of acid–base equilibria, chain conformations, and electrostatic interactions. In a recent work by Phillips et al., the Sawle-Ghosh theory was extended to include the effects of charge regulation.^36^ Similar to several other variational treatments of weak polyelectrolytes^37,38^ and polyampholytes,^39^ the approach of Phillips et al. was based on the mean-field assumption that the ionization state of weak acid and base groups does not depend on the position along the chain backbone. This assumption conflicts with previous theoretical and simulation results showing that the ionization profiles along weak polyelectrolytes are strongly nonuniform,^30,35,40,41^ because the ionization state of a particular residue depends on the local electrostatic environment experienced by that residue. Furthermore, while the chain conformation was treated systematically using the Edwards–Singh variational method, the effects of charge regulation were only included in an ad hoc manner. In order to go beyond these limitations, in this publication, we develop a sequence-specific variational theory for charge-regulating IDPs that explicitly models the monomer-specific ionization states. Moreover, our theory consistently treats both the ionization state and the chain conformation using the Edwards–Singh variational method.

## 2 Theory

### 2.1 Model

Following the Sawle–Ghosh theory,^2^ we model IDPs as polyampholytes described by the Edwards Hamiltonian,

βH=32l∫0Lds(dR(s)ds)2+l∫0Lds∫0sds′ω(s,s′)δ(R(s)−R(s′))+λBl2∫0Lds∫0sds′q(s)q(s′)exp(−κ|R(s)−R(s′)|)|R(s)−R(s′)| 1

which accounts for harmonic bonds, excluded volume interactions, and (screened) electrostatic interactions in an implicit solvent. *l* is the Kuhn length and *L* = *Nl* a measure for the contour length of the chain, with the number of monomers *N.* ω­(*s*, *s*′) is the dimensionless excluded volume parameter, which describes the (residue-specific) excluded volume interactions. λ_B_ ≡ *e* ^2^/4πε_0_ε_r_ *k*_B_ *T* is the Bjerrum length, which accounts for the dielectric constant ε_r_ of the implicit solvent and attains a value of 7.1 Å for aqueous solutions at room temperature. κ ≡ (8πλ_B_ *c*_NaCl_)^1/2^ is the Debye screening parameter, which depends on the salt concentration *c*_NaCl_ of the solution and determines the range of the screened electrostatic interactions. The function *q*(*s*) is the dimensionless backbone charge distribution along the chain, which takes values of *q*(*s*) = +1 and *q*(*s*) = −1 for ionized basic and acidic residues, respectively. We note that the model described by eq 1 cannot account for nonlinear effects like counterion condensation^42,43^ and related phenomena such as counterion release^44^ and the formation of transient dipoles,^36,45^ since the electrostatic interactions are described using the Debye–Hückel pair potential.

For a charge-regulating IDP, the model defined in eq 1 has to be extendend: the backbone charge profile *q*(*s*) becomes a fluctuating quantity due to the acid–base equilibria, which means that each realization of *q*(*s*) is assigned a statistical weight *W*[*q*(s)]. In the widely used constant-pH ensemble of Reed and Reed,^46^ this statistical weight is given by

2 $$W\left[q\left(s\right)\right]\propto \prod_{s}10^{\theta \left(s\right)\left(\mathrm{pH}-pK_{A}\left(s\right)\right)}$$

where the product runs over all weakly acidic and basic residues in the chain. Note that the overall normalization is omitted here, because it can always be determined a posteriori. p*K*_A_(*s*) is the p*K*_A_ value of residue *s* and θ­(*s*) is the corresponding degree of deprotonation, which is defined as

$$\theta \left(s\right)\equiv \{\begin{array}{ll} \left|q\left(s\right)\right|=-q\left(s\right) & \mathrm{for}\;\mathrm{acidic}\;\mathrm{residues}\\ 1-q\left(s\right) & \mathrm{for}\;\mathrm{basic}\;\mathrm{residues} \end{array}$$ 3

Thus, the statistical weight *W*[*q*(s)] can be written as

W[q(s)]∝exp(1l∫0Ldsθ(s)ln(10)[pH−pKA(s)])∝exp(−1l∫0Ldsq(s)ln(10)[pH−pKA(s)]) 4

The term in the exponential can be interpreted as a coupling of the backbone charge distribution to an external field ln(10)[pH–p*K*_A_(s)] that varies along the backbone. In the following, we treat this term as an additional contribution to the Hamiltonian, yielding the following total Hamiltonian of the weak polyampholyte chain:

βHt=32l∫0Lds(dR(s)ds)2+l∫0Lds∫0sds′ω(s,s′)δ(R(s)−R(s′))+λBl2∫0Lds∫0sds′q(s)q(s′)exp(−κ|R(s)−R(s′)|)|R(s)−R(s′)|+1l∫0Ldsq(s)ln(10)[pH−pKA(s)] 5

Given this Hamiltonian, the expectation values of observables *A* can be calculated by summing over chain conformations **R**(*s*) and backbone charge distributions *q*(*s*), with a statistical weight proportional to exp­(−β*H*_t_) for each microstate:

$$\left\langle A\right\rangle =\frac{\int DR\left(s\right)Dq\left(s\right)A\;\exp\left(-\beta H_{t}\right)}{\int DR\left(s\right)Dq\left(s\right)\exp\left(-\beta H_{t}\right)}$$ 6

In this equation, the path integrals over backbone charge distributions should be interpreted solely as a short-hand notation; in practice, we always discretize the chain before averaging over different charge states.

### 2.2 Variational Calculation

Because it is impossible to carry out the integrals in eq 6 exactly, we use the Edwards–Singh variational method^24,25^ to approximate the expectation values. Thus, we introduce an effective Hamiltonian,

7 $$\beta H_{r}=\frac{3}{2l_{r}}\int_{0}^{L}ds{\left(\frac{dR\left(s\right)}{ds}\right)}^{2}+\frac{1}{l}\int_{0}^{L}d\mathrm{sq}\left(s\right)\phi \left(s\right)$$

where *l*_r_ is the renormalized Kuhn length and ϕ­(*s*) is a residue-specific mean-field that acts on the charged residue at position *s* along the backbone. Here, the first term is the effective quadratic Hamiltonian introduced by Edwards and Singh.^24^ The idea behind this Hamiltonian is that the effect of interactions on the chain conformation can be described by a renormalized Kuhn length *l*_r_, i.e., by an effective bond stretching or compression (“uniform expansion model”). The second term accounts for charge regulation; similar trial Hamiltonians have previously been used in variational treatments of weak polyelectrolytes based on the Gibbs–Bogoliubov–Feynman inequality.^47,48^ Because the charge regulation term is linear in *q*(*s*), the charge-regulating residues are effectively decoupled and fluctuate independently in their local mean-fields ϕ­(*s*).

In the following, we treat the renormalized Kuhn length and the mean-fields as variational parameters, which we optimize to approximate the properties of the interacting system as closely as possible. Because we have to determine multiple variational parameters, we simultaneously apply the Edwards–Singh variational method to the squared end-to-end distance **R**_e_^2^ and the charge profile *q*(*u*), resulting in the equations

8 $${\left\langle R_{e}^{2}\right\rangle }_{r}{\left\langle \left(H_{r}-H_{t}\right)\right\rangle }_{r}={\left\langle R_{e}^{2}\left(H_{r}-H_{t}\right)\right\rangle }_{r}$$

9 $${\left\langle q\left(u\right)\right\rangle }_{r}{\left\langle \left(H_{r}-H_{t}\right)\right\rangle }_{r}={\left\langle q\left(u\right)\left(H_{r}-H_{t}\right)\right\rangle }_{r}$$

Evaluating the expectation values and adopting a discrete notation (please refer to the Supporting Information (SI) for detailed calculations), we arrive at a set of coupled, self-consistent equations for the renormalized Kuhn length and the residue-specific mean-fields. Note that in the discrete notation, the position along the backbone is described by a discrete index. The renormalized Kuhn length is obtained from the equation

$$\begin{array}{c} N\left(\frac{1}{x}-1\right)+{\left(\frac{3}{2\pi }\right)}^{3/2}\frac{1}{x^{5/2}}\overset{N}{\underset{m=2}{\sum }}\overset{m-1}{\underset{n=1}{\sum }}\frac{\omega_{\mathrm{mn}}}{{\left(m-n\right)}^{1/2}}\\ +\frac{2\lambda_{B}}{9\pi l}\frac{1}{x^{3/2}}\overset{N}{\underset{m=2}{\sum }}\overset{m-1}{\underset{n=1}{\sum }}\left\langle q_{m}\right\rangle \left\langle q_{n}\right\rangle {\left(m-n\right)}^{2}A_{\mathrm{mn}}\left(x,\kappa l\right)=0 \end{array}$$ 10

where *x* = *l*_r_/*l* and the matrix elements *A*_*mn*_(*x*, *κl*) are defined in eq (S12) in the SI. For the mean-fields ϕ_*i*_, we get a total of *N* equations,

$$\phi_{i}=\ln\left(10\right)\left(\mathrm{pH}-pK_{A,i}\right)+\frac{2\lambda_{B}}{\pi l}\overset{N}{\underset{\begin{array}{l} m=1\\ m\neq i \end{array}}{\sum }}\left\langle q_{m}\right\rangle J_{\mathrm{mi}}\left(x,\kappa l\right)$$ 11

with the matrix elements *J*_*mi*_(*x*, *κl*) defined in eq (S29) in the SI. The average charges appearing in eqs 10 and 11 are self-consistently determined by the mean-fields:

$$\left\langle q_{m}\right\rangle =\{\begin{array}{ll} -1/\left(1+\exp\left(-\phi_{m}\right)\right) & \mathrm{for}\;\mathrm{acidic}\;\mathrm{residues}\\ 1/\left(1+\exp\left(\phi_{m}\right)\right) & \mathrm{for}\;\mathrm{basic}\;\mathrm{residues} \end{array}$$ 12

The main equations of our theory (eqs 10 to 12) have a straightforward physical interpretation. Equation 10 determines the chain conformation, which arises due to a balance between bonded interactions, excluded volume interactions, and electrostatic interactions. This equation has exactly the same form as the equation derived by Sawle and Ghosh for quenched IDPs,^2^ the only difference being that in our theory the charges are not fixed, but have to be calculated self-consistently using the mean-fields. Equation 11 for the mean-fields in combination with eq 12 has the same mathematical structure as the mean-field theory for spin systems, with two distinct contributions to the mean-fields. The first term in eq 11, ln(10)­(pH–p*K*_A,*i*_), describes the ideal/one-body contribution to charge regulation, analogous to an external field in spin systems. The second term in eq 11, $\frac{2\lambda_{B}}{\pi l}\overset{N}{\underset{\begin{array}{l} m=1\\ m\neq i \end{array}}{\sum }}\left\langle q_{m}\right\rangle J_{\mathrm{mi}}\left(x,\kappa l\right)$ , describes the residue–residue interactions at the mean-field level and can also be interpreted as the p*K*_A_ shift induced by the electrostatic interactions. Notably, this equation accounts for the effective Gaussian chain structure, which is contained in the coupling matrix *J* through the value of *x* and controls the effective coupling strength between residues. We note that eq 11 corresponds to a mean-field version of the theory proposed by Zhou to treat acid–base equilibria in unfolded proteins.^49,50^ However, this earlier theory treated the effective Kuhn length as an empirical input parameter. In contrast, our theory requires no a priori assumption regarding the value of *l_r_*, because it (approximately) captures the complex coupling between ionization states and chain conformations that is characteristic of weak polyelectrolytes and polyampholytes.

Before solving the derived equations numerically for specific systems, it is instructive to briefly consider a few simple limiting cases of our theory:

1. Strong screening limit: for *κl* → ∞, the matrices *A* and *J* vanish. Therefore, the chain structure is solely determined by the excluded volume interactions, and the charged residues ionize according to the Henderson–Hasselbalch equation.
2. Weak coupling limit: the same behavior as in the strong screening limit is also expected in the weak coupling limit λ_B_/*l* → 0.
3. Extreme pH limit: for (pH–p*K*_A,*i*_)→ ± ∞, all residues are fully ionized or neutral. Thus, the theory reduces to the special case of a strong polyampholyte, i.e., the Sawle–Ghosh theory for quenched IDPs.^2^
4. Quenched chain conformation limit: if the renormalized Kuhn length is set to a fixed value and not determined using eq 10, our theory is equivalent to the mean-field approximation of the theory developed by Zhou.^49,50^

### 2.3 Numerical Approach

Because eqs 10 to 12 are highly nonlinear, they cannot be solved in closed form. To solve these equations numerically, we use the following algorithm:

1. Make an initial guess for the mean-fields ϕ_*i*_. A reasonable choice is the ideal value ϕ_*i*_ = ln(10)­(pH–p*K*_A,*i*_) calculated using the Henderson–Hasselbalch equation. Use eq 12 to calculate the average charges ⟨*q*_*i*_⟩ based on this initial guess.
2. Insert the average charges into eq 10 and solve the equation numerically for *x*.
3. Use the average charges ⟨*q*_*i*_⟩ and the determined value of *x* to calculate the right-hand side of eq 11. Update the mean-fields using the simple mixing algorithm^51^
ϕi←(1−ζ)ϕi+ζ[ln(10)(pH−pKA,i)+2λBπl∑m=1m≠iN⟨qm⟩Jmi(x,κl)] 13
  with the mixing parameter ζ∈(0,1]. In the following, we use ζ = 0.5.
4. Use eq 12 to calculate the average charges ⟨*q*_*i*_⟩ for the updated mean-fields. Return to step 2 and repeat.

This loop is repeated until the quantities ⟨*q*_*i*_⟩ and *x* have converged within the desired numerical tolerance. In the following, we use a tolerance of 10^–10^. At this numerical precision, on a standard laptop the equations can be solved in seconds for IDPs of moderate length.

In principle, the nonlinear equations of our theory could allow for multiple solutions, which would correspond to various (meta)­stable states. However—for the model and parameters studied here—we never find the typical signatures of coexisting states, i.e., jumps in the ionization/charge or end-to-end distance. Moreover, solving the equations with various randomly chosen initial conditions for *x* and the mean-fields always results in the same, unique ionization state and end-to-end distance. We hypothesize that multiple distinct solutions of our equations might emerge in the case where hydrophobic interactions are taken into account,^15,52^ i.e., for a negative second virial coefficient. Treating this case would, however, require the inclusion of repulsive three-body interactions in the model to stabilize the polymer.

## 3 Results

### 3.1 Weak Polyelectrolyte

As a first application of our theory, we consider a weak polyacid chain with *N* = 30 monomers in a monovalent salt solution (the salt concentration and valency are encoded in the Debye screening length). We set the Bjerrum length to a value of λ_B_= 7.1 Å, the monomer p*K*_A_ value to p*K*_A_ = 4.0 and, following Sawle and Ghosh,^2^ the Kuhn length to a value of *l* = 5.8 Å. Moreover, to focus solely on electrostatic effects, we do not consider excluded volume interactions (ω = 0).

In Figure 1a, we show the degree of ionization of the weak polyacid for different salt concentrations. Similar to previous simulation studies, the theory predicts that the degree of ionization is always lower than the ideal Henderson–Hasselbalch result, this is the so-called “polyelectrolyte effect.”^53^ The suppressed ionization is caused by strong intramolecular repulsions, which our theory encodes in the matrix *J*. Furthermore, the difference to the ideal curve is correctly predicted to become smaller with increasing salt concentration due to the stronger Debye screening. In addition to the ionization state, our theory also allows us to calculate the end-to-end distance, which is shown in Figure 1b. As expected, the polyelectrolyte chain swells with increasing pH value, concomitant with increasing ionization. With decreasing salt concentration, swelling becomes more pronounced because there is less screening of the electrostatic repulsion among the monomers. In addition, the onset of swelling gradually shifts to higher pH values, which is a consequence of the shifted ionization response.

**Figure 1:**
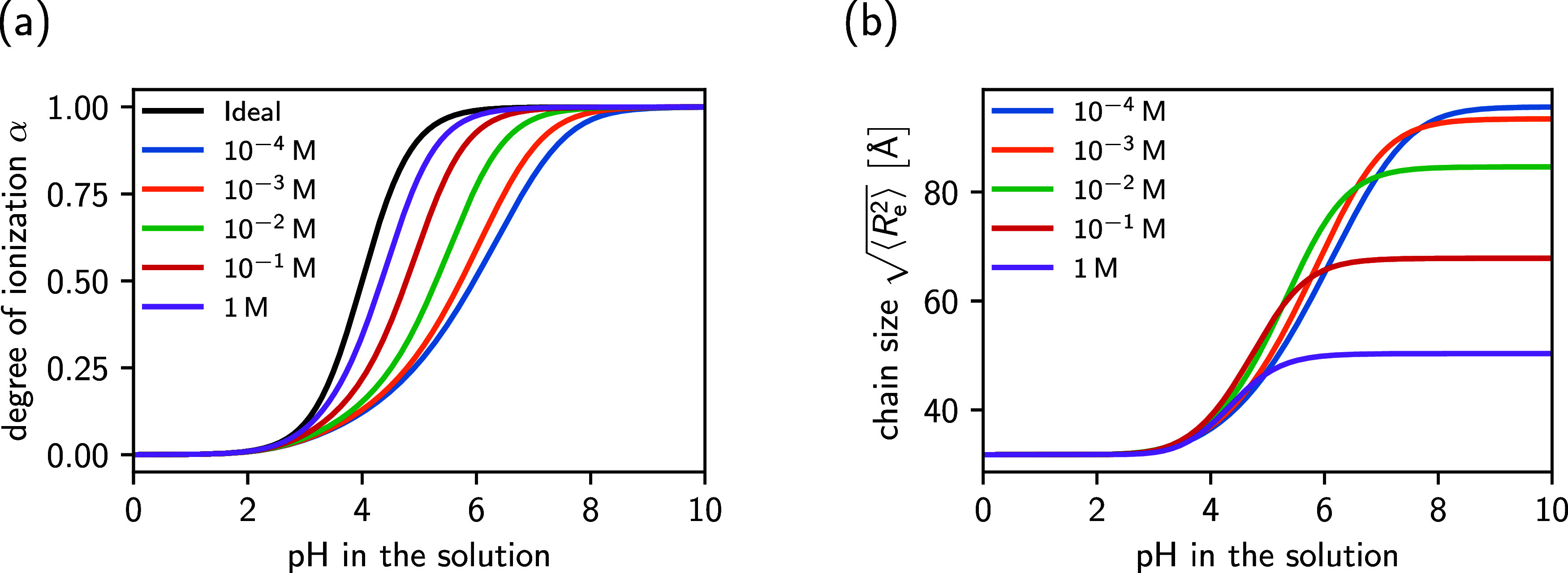
Degree of ionization (a) and end-to-end distance (b) of a weak polyacid as a function of pH. The results were obtained for a chain of length *N* = 30, with a Kuhn length of *l* = 5.8 Å and a monomer p*K*_A_ value of p*K*_A_ = 4.0. Curves of different colors correspond to different bulk salt concentrations indicated in the legend.

Lastly, we can also predict the monomer-resolved ionization profile along the polyacid backbone, which is shown in Figure 2 for a salt concentration of 10^–2^ M. In qualitative agreement with previous studies, the degree of ionization increases toward the ends of the chain.^35,40,41^ This effect arises because the number of nearby interaction partners for residues near the chain ends is smaller than in the center, leading to a lower electrostatic penalty associated with the ionized state.

**Figure 2:**
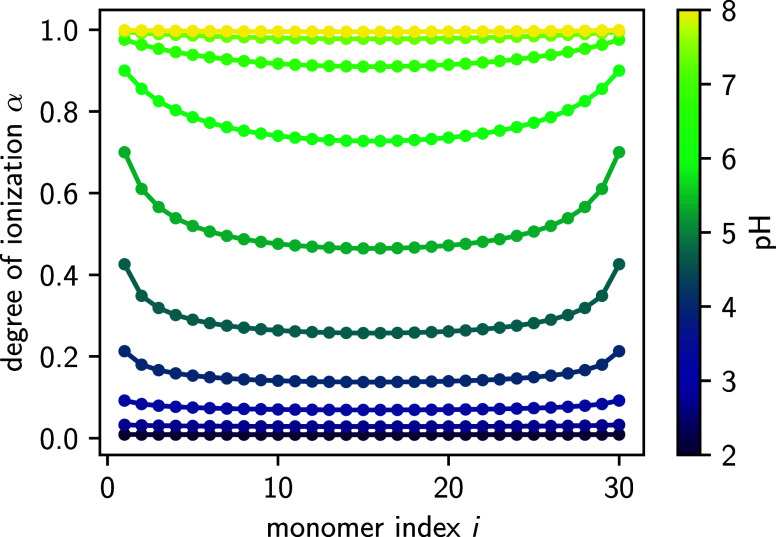
Monomer-resolved degree of ionization along a weak polyacid for different pH values and a salt concentration of 10^–2^ M. The results were obtained for a chain of length *N* = 30, with a Kuhn length of *l* = 5.8 Å and a monomer p*K*_A_ value of p*K*_A_ = 4.0. The lines serve only as a guide for the eyes.

### 3.2 Charge-Regulating IDPs

As a second example, we consider sequence variants of the polypeptide (EK)_25_, which contains Glutamic acid (E, a weak acid) and Lysine (K, a weak base). The 30 sequence variants used in the following, shown in Figure 3, were first studied in the seminal work of Das and Pappu^54^ and are listed in order of increasing blockiness. Note that the blockiness can be quantified using various, mutually correlated measures such as the charge segregation metric^54^ or the sequence charge decoration metric.^2^ Because the 30 sequence variants in Figure 3 span a wide range of blockiness, they have become a popular model system for studying sequence effects in IDPs.^2,19^ For our calculations, we set the Bjerrum length to a value of λ_B_= 7.1 Å, the salt concentration to *c*_NaCl_= 0.015 M and, following Sawle and Ghosh,^2^ the Kuhn length to a value of *l* = 5.8 Å. The sequence-specific excluded volume parameters ω are also taken from the earlier work.^2^ We use the p*K*_A_ values reported by Nozaki and Tanford^55^ for amino acids (p*K*_A_ = 4.4 for Glutamic acid and p*K*_A_ = 10.4 for Lysine).

**Figure 3:**
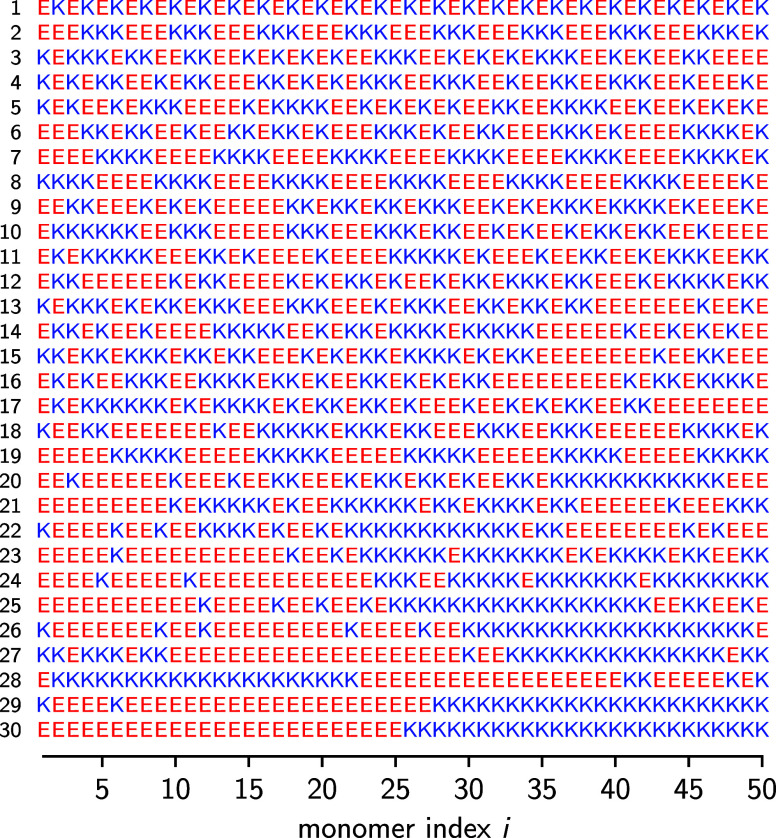
Sequence variants of the polypeptide (EK)_25_ originally studied by Das and Pappu.^54^ With increasing sequence number, the sequences become blockier.

In Figure 4a, we show the predicted net charge of selected sequence variants as a function of the pH value (a corresponding graph containing all sequences is provided in Figure S1 in the SI). Crucially, we observe pronounced sequence effects on the ionization response, i.e., different sequences strongly differ in their net charge at the same pH value. As a general trend, we can identify that sequences with low blockiness (corresponding to low sequence numbers) exhibit a wide range of pH values where the peptide is practically net neutral. Compared with the ideal Henderson–Hasselbalch prediction, this plateau is broadened. For blockier sequences, the plateau becomes narrower, eventually vanishing completely. Overall, this behavior is similar to the ionization response of homogeneous and patchy nanoparticles, which was recently studied using coarse-grained simulations.^56^

**Figure 4:**
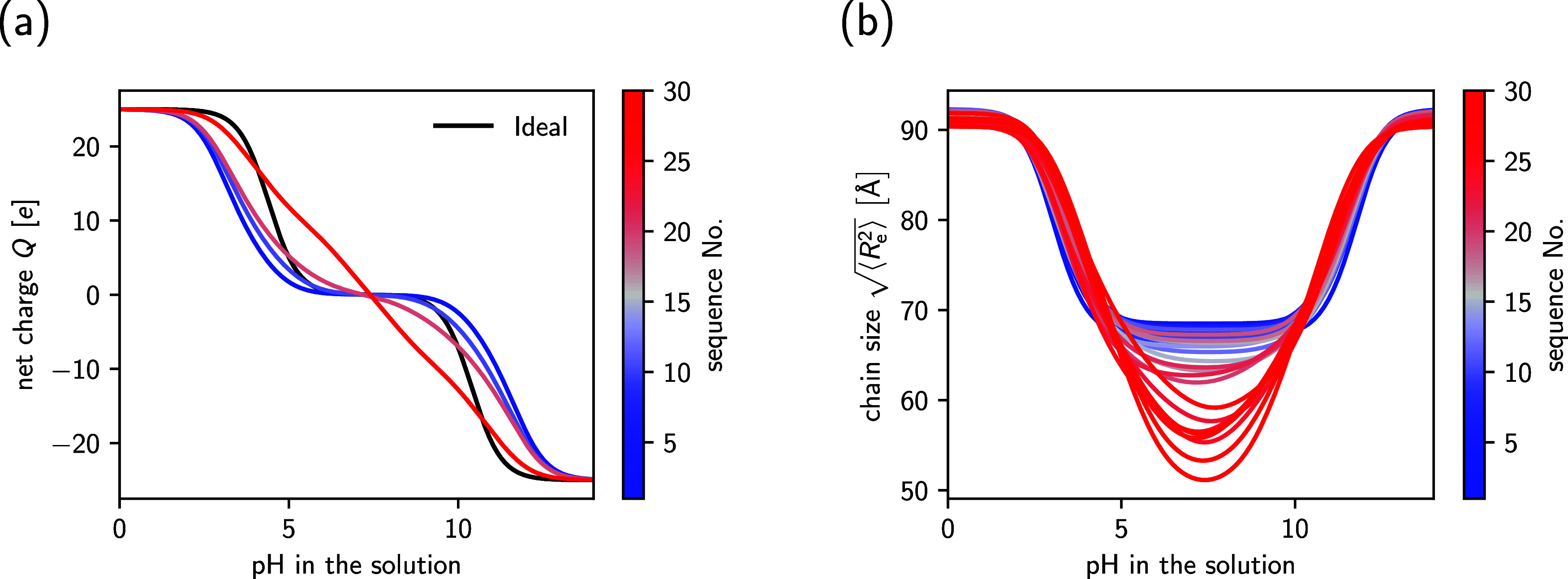
Net charge (a) and end-to-end distance (b) of various IDPs with composition (EK)_25_ and different sequences as a function of the pH value. Larger sequence numbers correspond to blockier sequences. For better visibility, only the sequences 1, 10, 20, and 30 are shown in subfigure (a).

To better understand the predicted ionization behavior, we examine the monomer-resolved charge profile along various sequence variants (Figure 5). Because the behavior should be roughly symmetrical around the isoelectric point of pI ≈ 7.0, we focus on the regime pH ≥ 7.0. For the strictly alternating sequence variant 1 (Figure 5a–c), the charge state of an amino acid is almost independent of its position along the polymer backbone for all pH values. This behavior emerges, because the ionization equilibrium is dominated by favorable electrostatic interactions with neighboring monomers. Only at the chain ends can some variation be observed for pH = 10.0: the terminal Lysine residue at position 50 is slightly less ionized than the others, because it has one oppositely charged neighbor instead of two. The observed ionization structure can be directly related to our earlier observations regarding the net charge: the zwitterionic state of the alternating sequence is highly stabilized by favorable electrostatic interactions between nearby monomers, which leads to the broad plateau observed in Figure 4a. As the blockiness of the sequences increases, we observe a more interesting ionization structure: similar to the weak polyacid in Figure 2, the charge profiles of longer blocks have a characteristic U-shape, with a higher ionization toward the ends. The origin of the observed U-shape is twofold: for blocks located at a chain end (see sequence No. 30), the enhanced ionization toward the chain end is again caused by the lower number of possible interaction partners that would suppress highly ionized states. In contrast, chain-internal block boundaries (see sequence Nos. 10, 20 and 30) exhibit an enhanced ionization due to favorable electrostatic interactions with the oppositely charged neighboring block. Because repulsive electrostatic interactions within the blocks increasingly dominate the ionization behavior as the block length increases, blockier sequences exhibit a much smaller range of pH values where the IDP is overall neutral, leading to the vanishing of the plateau observed in Figure 4a.

**Figure 5:**
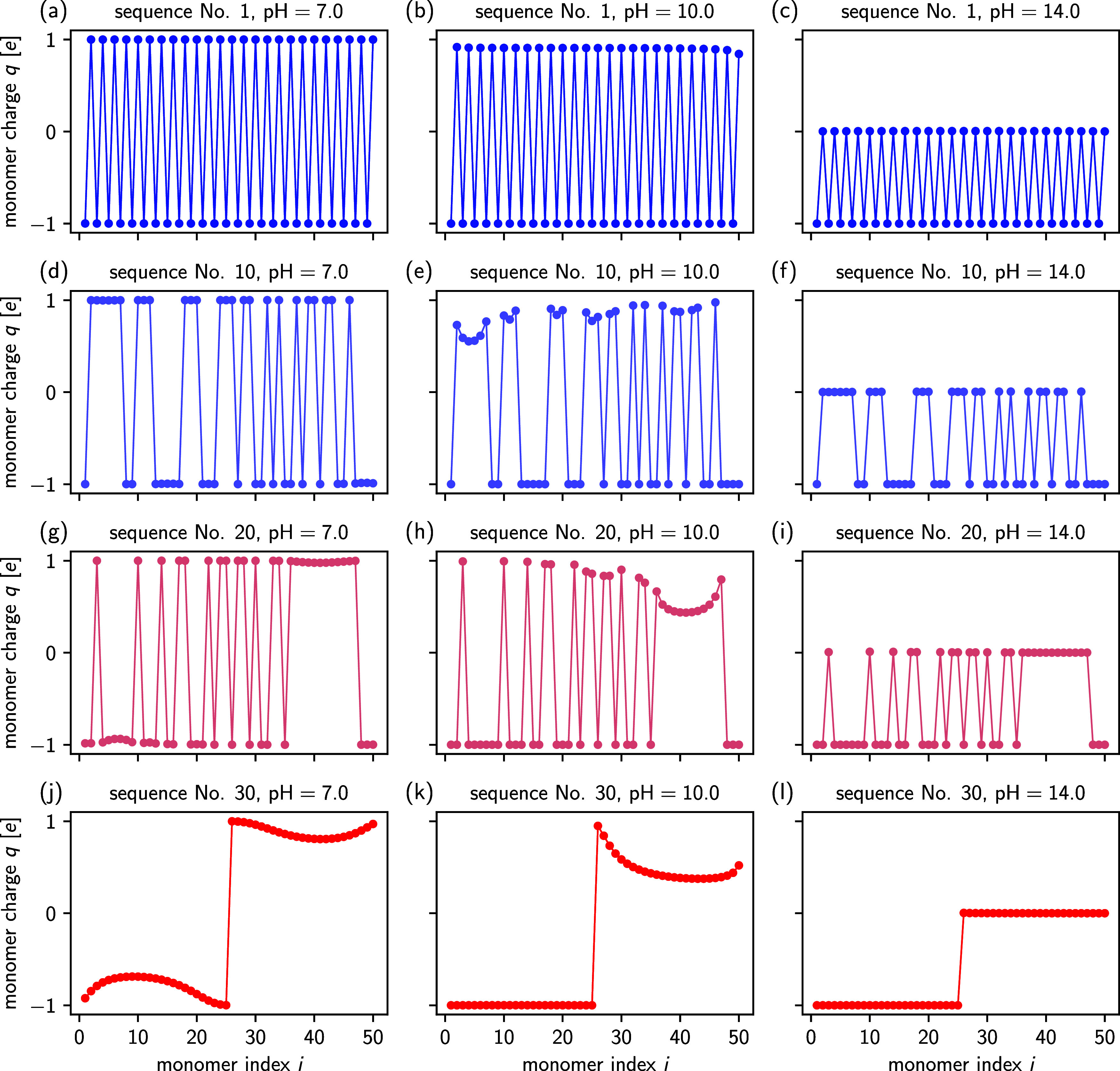
Charge profiles along the (EK)_25_ sequence variants 1 (a–c), 10 (d–f), 20 (g–i) and 30 (j–l). The three columns correspond to pH values of 7.0 (left column), 10.0 (middle column) and 14.0 (right column). Sequences are color-coded identically to Figure 4. The lines serve only as a guide for the eyes.

Finally, we investigate the end-to-end distances for different sequence variants (Figure 4b). Similarly to earlier studies on weak polyampholytes,^39,57,58^ we observe that the IDPs are swollen at extreme pH values, where the chains acquire a significant net charge. In contrast, they adopt more compact conformations around the isoelectric point, where they are almost net neutral. Because the ionization state is coupled to the chain conformation, we also observe strong sequence effects on the pH-dependent end-to-end distance. In general, blockier sequences strongly collapse near the isolelectric point, but the pH range in which the IDP remains compact is relatively narrow. In contrast, the collapse of well-mixed sequences is less pronounced, but the IDP stays compact over a broad range of pH values.

## 4 Conclusion and Outlook

To summarize, we have extended the Sawle–Ghosh theory for intrinsically disordered proteins^2^ to explicitly include acid–base equilibria of amino acid residues. In particular, unlike previous treatments,^36–39^ the proposed theory does not assume a uniform ionization state of residues along the sequence. Our main result is a set of coupled algebraic equations for the renormalized Kuhn length and the residue-specific mean-fields. We discussed a few limiting cases of these equations, which underline the internal consistency of our theory and connect our results to earlier studies. To solve the full set of equations, we proposed a simple numerical scheme.

As a simple test case, we calculated the conformation and ionization states of a weak polyacid at various pH values and salt concentrations. These results showed that the theory correctly predicts the “polyelectrolyte effect” and its salt dependence, as well as the pH-responsive swelling of the weak polyelectrolyte. Furthermore, the nonuniform ionization profile along the chain backbone—with an increasing ionization toward the chain ends—is also captured. As a second test case, we considered 30 sequence variants of the polypeptide (EK)_25_. For this system, we found strong sequence effects on the ionization, with well-mixed sequences exhibiting a broad pH range in which the polypeptide was net neutral, while blockier sequences exhibited a steeper ionization response. The predicted ionization behavior could be rationalized by analyzing the monomer-resolved charge profile of various IDP sequences. Moreover, we also observed pronounced sequence effects on the swelling behavior of the chains.

In the future, it will be interesting to test our variational theory against coarse-grained or atomistic simulations that explicitly account for charge regulation, which can be carried out using a range of state-of-the-art methods.^30,46,59–62^ Furthermore, there are also several extensions of our theory that could be explored. For example, following previous work, it should also be possible to include hydrophobic interactions in the model.^15^ Finally, it would be worthwhile to compare the predictions of our theory with the results of titration experiments.

## Supplementary Material



## Notes

Figure S1: Net charge of various IDPs with composition (EK)_25_; a detailed derivation of the theory (PDF)
