# Supplementary material for: A Sequence-Specific Theory for Charge-Regulating IDPs
Source: J Phys Chem B. 2026 Apr 13;130(16):4325–32. doi: 10.1021/acs.jpcb.6c00655 (PMC13112352; doi:10.1021/acs.jpcb.6c00655)
Supplement: Supplementary file 1 [file jp6c00655_si_001.pdf]

Supporting Information: A Sequence-Specific Theory for Charge-Regulating IDPs

David Beyer,*,† Christian Holm,*,† and Zhen-Gang Wang*,‡

†*Institute for Computational Physics, University of Stuttgart, D-70569 Stuttgart, Germany*

‡*Division of Chemistry and Chemical Engineering, California Institute of Technology, Pasadena, California 91125, USA*

E-mail: dbeyer@icp.uni-stuttgart.de; holm@icp.uni-stuttgart.de; zgw@caltech.edu

1 Additional Figures

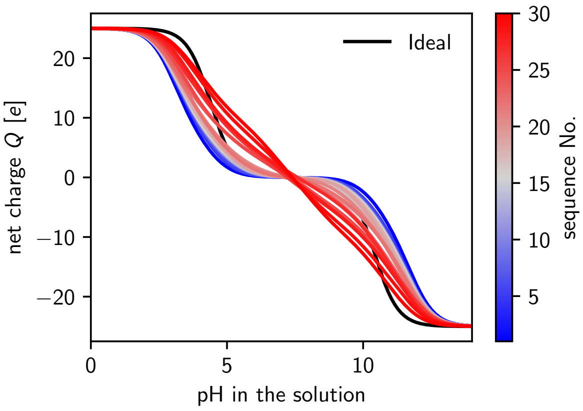

Figure S1: Net charge of various IDPs with composition $(EK)_{25}$ and different sequences as a function of the pH value. Larger sequence numbers correspond to blockier sequences.

2 Detailed Calculations

Here, we derive explicit expressions for Equations (8) and (9) in the main text, which ultimately result in the main equations of our theory (Equations (10) to (12)). Many of the calculations have previously been carried out in an almost identical form for quenched IDPs by Sawle and Ghosh[S1] and require only minor modifications. In the following, it will be useful to note that expectation values $\langle ... \rangle_{\mathrm{r}}$ are double averages, i.e. an average $\langle ... \rangle_{\mathbf{R}}$ over chain conformations $\mathbf{R}(s)$ and an average $\langle ... \rangle_{q}$ over ionization states $q(s)$:

$$\langle ... \rangle_{\mathrm{r}} = \langle \langle ... \rangle_{\mathbf{R}} \rangle_{q} \,. \tag{S1}$$

Because the trial Hamiltonian given in Equation (7) does not couple chain conformations and ionization states, expectation values of products $A = a\left[\mathbf{R}(s)\right] b\left[q(s)\right]$ conveniently factorize:

$$\langle A \rangle_{\mathrm{r}} = \langle a \rangle_{\mathbf{R}} \langle b \rangle_{q} \,. \tag{S2}$$

In the following, we set $\beta = 1$ to somewhat simplify the equations.

2.1 Variational Calculation of Chain Size

2.1.1 Preliminaries

We first derive an explicit expression for Equation 8, which involves the end-to-end distance

$$\mathbf{R}_{\mathrm{e}}^2 = \left(\mathbf{R}(L) - \mathbf{R}(0)\right)^2 . \tag{S3}$$

To make the lengthy calculation more manageable, we consider the contributions arising from different physical effects separately and combine them at the very end. $\langle (H_{\mathrm{r}} - H_{\mathrm{t}}) \rangle_{\mathrm{r}}$ can be divided into different contributions accounting for harmonic bonds, excluded volume

interactions, electrostatic interactions, and charge regulation:

$$
\begin{aligned}
\left\langle (H_{\mathrm{r}} - H_{\mathrm{t}}) \right\rangle_{\mathrm{r}} = & \left\langle \frac{3}{2} \left(\frac{1}{l_{\mathrm{r}}} - \frac{1}{l} \right) \int_0^L \mathrm{d}s \left(\frac{\mathrm{d}\mathbf{R}(s)}{\mathrm{d}s} \right)^2 \right\rangle_{\mathrm{r}} \\
& - \left\langle l \int_0^L \mathrm{d}s \int_0^s \mathrm{d}s' \omega(s,s') \delta\left(\mathbf{R}(s) - \mathbf{R}(s') \right) \right\rangle_{\mathrm{r}} \\
& - \left\langle \frac{\lambda_{\mathrm{B}}}{l^2} \int_0^L \mathrm{d}s \int_0^s \mathrm{d}s' q(s) q(s') \frac{\exp\left(-\kappa \left| \mathbf{R}(s) - \mathbf{R}(s') \right|\right)}{\left| \mathbf{R}(s) - \mathbf{R}(s') \right|} \right\rangle_{\mathrm{r}} \\
& + \left\langle \frac{1}{l} \int_0^L \mathrm{d}s\, q(s) \left(\phi(s) - \ln(10) \left[\mathrm{pH} - \mathrm{p}K_{\mathrm{A}}(s) \right] \right) \right\rangle_{\mathrm{r}} .
\end{aligned}
\tag{S4}
$$

Similarly, $\left\langle \mathbf{R}_{\mathrm{e}}^2 \left(H_{\mathrm{r}} - H_{\mathrm{t}} \right) \right\rangle_{\mathrm{r}}$ can be split:

$$
\begin{aligned}
& \left\langle \mathbf{R}_{\mathrm{e}}^2 \left(H_{\mathrm{r}} - H_{\mathrm{t}} \right) \right\rangle_{\mathrm{r}} \\
= & \left\langle \left(\mathbf{R}(L) - \mathbf{R}(0) \right)^2 \frac{3}{2} \left(\frac{1}{l_{\mathrm{r}}} - \frac{1}{l} \right) \int_0^L \mathrm{d}s \left(\frac{\mathrm{d}\mathbf{R}(s)}{\mathrm{d}s} \right)^2 \right\rangle_{\mathrm{r}} \\
& - \left\langle \left(\mathbf{R}(L) - \mathbf{R}(0) \right)^2 l \int_0^L \mathrm{d}s \int_0^s \mathrm{d}s' \omega(s,s') \delta\left(\mathbf{R}(s) - \mathbf{R}(s') \right) \right\rangle_{\mathrm{r}} \\
& - \left\langle \left(\mathbf{R}(L) - \mathbf{R}(0) \right)^2 \frac{\lambda_{\mathrm{B}}}{l^2} \int_0^L \mathrm{d}s \int_0^s \mathrm{d}s' q(s) q(s') \frac{\exp\left(-\kappa \left| \mathbf{R}(s) - \mathbf{R}(s') \right|\right)}{\left| \mathbf{R}(s) - \mathbf{R}(s') \right|} \right\rangle_{\mathrm{r}} \\
& + \left\langle \left(\mathbf{R}(L) - \mathbf{R}(0) \right)^2 \frac{1}{l} \int_0^L \mathrm{d}s\, q(s) \left(\phi(s) - \ln(10) \left[\mathrm{pH} - \mathrm{p}K_{\mathrm{A}}(s) \right] \right) \right\rangle_{\mathrm{r}} .
\end{aligned}
\tag{S5}
$$

We now proceed to evaluate the different expectation values.

2.1.2 Harmonic Bonds

The harmonic bond terms do not involve $q(s)$ and therefore reduce to expectation values over different chain conformations. The result can thus be taken directly from the paper by

Sawle and Ghosh:[S1]

$$\begin{aligned}
&\left\langle (\mathbf{R}(L)-\mathbf{R}(0))^2 \frac{3}{2}\left(\frac{1}{l_\mathrm{r}}-\frac{1}{l}\right)\int_0^L \mathrm{d}s\left(\frac{\mathrm{d}\mathbf{R}(s)}{\mathrm{d}s}\right)^2\right\rangle_\mathrm{r}\\
&-\left\langle (\mathbf{R}(L)-\mathbf{R}(0))^2\right\rangle_\mathrm{r}\left\langle \frac{3}{2}\left(\frac{1}{l_\mathrm{r}}-\frac{1}{l}\right)\int_0^L \mathrm{d}s\left(\frac{\mathrm{d}\mathbf{R}(s)}{\mathrm{d}s}\right)^2\right\rangle_\mathrm{r}\\
=&\left\langle (\mathbf{R}(L)-\mathbf{R}(0))^2 \frac{3}{2}\left(\frac{1}{l_\mathrm{r}}-\frac{1}{l}\right)\int_0^L \mathrm{d}s\left(\frac{\mathrm{d}\mathbf{R}(s)}{\mathrm{d}s}\right)^2\right\rangle_\mathbf{R}\\
&-\left\langle (\mathbf{R}(L)-\mathbf{R}(0))^2\right\rangle_\mathbf{R}\left\langle \frac{3}{2}\left(\frac{1}{l_\mathrm{r}}-\frac{1}{l}\right)\int_0^L \mathrm{d}s\left(\frac{\mathrm{d}\mathbf{R}(s)}{\mathrm{d}s}\right)^2\right\rangle_\mathbf{R}\\
=&l_\mathrm{r}^2 L\left(\frac{1}{l_\mathrm{r}}-\frac{1}{l}\right).
\end{aligned} \tag{S6}$$

Discretizing the continuous chain and introducing the abbreviation $x = l_\mathrm{r}/l$, we arrive at[S1]

$$l_\mathrm{r}^2 L\left(\frac{1}{l_\mathrm{r}}-\frac{1}{l}\right) \xrightarrow{\text{discretize}} l_\mathrm{r}^2 l N\left(\frac{1}{l_\mathrm{r}}-\frac{1}{l}\right) = l_\mathrm{r}^2 N\left(\frac{1}{x}-1\right). \tag{S7}$$

2.1.3 Excluded Volume Interactions

The excluded volume terms likewise do not depend on the backbone charge and can also be taken from the paper by Sawle and Ghosh:[S1]

$$\begin{aligned}
&\left\langle (\mathbf{R}(L)-\mathbf{R}(0))^2\, l\int_0^L \mathrm{d}s\int_0^s \mathrm{d}s'\omega(s,s')\delta\left(\mathbf{R}(s)-\mathbf{R}(s')\right)\right\rangle_\mathrm{r}\\
&-\left\langle (\mathbf{R}(L)-\mathbf{R}(0))^2\right\rangle_\mathrm{r}\left\langle l\int_0^L \mathrm{d}s\int_0^s \mathrm{d}s'\omega(s,s')\delta\left(\mathbf{R}(s)-\mathbf{R}(s')\right)\right\rangle_\mathrm{r}\\
=&\left\langle (\mathbf{R}(L)-\mathbf{R}(0))^2\, l\int_0^L \mathrm{d}s\int_0^s \mathrm{d}s'\omega(s,s')\delta\left(\mathbf{R}(s)-\mathbf{R}(s')\right)\right\rangle_\mathbf{R}\\
&-\left\langle (\mathbf{R}(L)-\mathbf{R}(0))^2\right\rangle_\mathbf{R}\left\langle l\int_0^L \mathrm{d}s\int_0^s \mathrm{d}s'\omega(s,s')\delta\left(\mathbf{R}(s)-\mathbf{R}(s')\right)\right\rangle_\mathbf{R}\\
=&-\frac{l_\mathrm{r}^2 l}{3}\left(\frac{1}{2\pi}\right)^{3/2}\left(\frac{3}{l_\mathrm{r}}\right)^{5/2}\int_0^L \mathrm{d}s\int_0^s \mathrm{d}s'\frac{\omega(s,s')}{(s-s')^{1/2}}.
\end{aligned} \tag{S8}$$

Discretizing the continuous chain and introducing the indices $m = s/l$, $n = s'/l$, we obtain[S1]

$$
\begin{aligned}
& - \frac{l_\mathrm{r}^2 l}{3} \left(\frac{1}{2\pi}\right)^{3/2} \left(\frac{3}{l_\mathrm{r}}\right)^{5/2} \int_0^L \mathrm{d}s \int_0^s \mathrm{d}s' \frac{\omega(s,s')}{(s-s')^{1/2}} \\
\xrightarrow{\text{discretize}} & - \frac{l_\mathrm{r}^2 l^{5/2}}{3} \left(\frac{1}{2\pi}\right)^{3/2} \left(\frac{3}{l_\mathrm{r}}\right)^{5/2} \sum_{m=2}^{N} \sum_{n=1}^{m-1} \frac{\omega_{mn}}{(m-n)^{1/2}} \\
= & - l_\mathrm{r}^2 \left(\frac{3}{2\pi}\right)^{3/2} \frac{1}{x^{5/2}} \sum_{m=2}^{N} \sum_{n=1}^{m-1} \frac{\omega_{mn}}{(m-n)^{1/2}} .
\end{aligned} \tag{S9}
$$

Here, the double sum runs over all pairs of monomers. Note that spurious self-interactions are explicitly not included.

2.1.4 Electrostatic Interactions

For the terms involving the Debye-Hückel potential, we take the result of Sawle and Ghosh[S1] and additionally average over ionization states:

$$
\begin{aligned}
& \left\langle (\mathbf{R}(L) - \mathbf{R}(0))^2 \frac{\lambda_\mathrm{B}}{l^2} \int_0^L \mathrm{d}s \int_0^s \mathrm{d}s' q(s) q(s') \frac{\exp\left(-\kappa \left|\mathbf{R}(s) - \mathbf{R}(s')\right|\right)}{\left|\mathbf{R}(s) - \mathbf{R}(s')\right|} \right\rangle_\mathrm{r} \\
& - \left\langle (\mathbf{R}(L) - \mathbf{R}(0))^2 \right\rangle_\mathrm{r} \left\langle \frac{\lambda_\mathrm{B}}{l^2} \int_0^L \mathrm{d}s \int_0^s \mathrm{d}s' q(s) q(s') \frac{\exp\left(-\kappa \left|\mathbf{R}(s) - \mathbf{R}(s')\right|\right)}{\left|\mathbf{R}(s) - \mathbf{R}(s')\right|} \right\rangle_\mathrm{r} \\
= & - l_\mathrm{r}^2 \frac{4\pi\lambda_\mathrm{B}}{9 l^2} \int_0^L \mathrm{d}s \int_0^s \mathrm{d}s' \left\langle q(s) q(s') \right\rangle_q (s-s')^2 \times \\
& \times \int \frac{\mathrm{d}^3 k \, k^2}{(2\pi)^3 (k^2 + \kappa^2)} \exp\left(-\frac{k^2 l_\mathrm{r} \left|s - s'\right|}{6}\right) \\
= & - l_\mathrm{r}^2 \frac{4\pi\lambda_\mathrm{B}}{9 l^2} \int_0^L \mathrm{d}s \int_0^s \mathrm{d}s' \left\langle q(s) q(s') \right\rangle_q (s-s')^2 \frac{1}{2\pi^2} \times \\
& \times \left[\frac{\pi^{1/2}}{4} \left(\frac{6}{l_\mathrm{r} \left|s - s'\right|}\right)^{3/2} - \frac{\pi^{1/2}}{2} \kappa^2 \left(\frac{6}{l_\mathrm{r} \left|s - s'\right|}\right)^{1/2} \right. \\
& \left. + \frac{\pi}{2} \kappa^3 \exp\left(\frac{\kappa^2 l_\mathrm{r} \left|s - s'\right|}{6}\right) \mathrm{erfc}\left(\sqrt{\frac{\kappa^2 l_\mathrm{r} \left|s - s'\right|}{6}}\right) \right] .
\end{aligned} \tag{S10}
$$

Discretizing the chain, introducing the abbreviation $x = l_\mathrm{r}/l$ and introducing the indices $m = s/l$, $n = s'/l$, we arrive at[S1]

$$
\begin{aligned}
&- l_\mathrm{r}^2 \frac{4\pi\lambda_\mathrm{B}}{9l^2} \int_0^L \mathrm{d}s \int_0^s \mathrm{d}s' \left\langle q(s)q(s')\right\rangle_q \left(s-s'\right)^2 \frac{1}{2\pi^2} \times \\
&\times \left[\frac{\pi^{1/2}}{4} \left(\frac{6}{l_\mathrm{r}\left|s-s'\right|} \right)^{3/2} - \frac{\pi^{1/2}}{2}\kappa^2 \left(\frac{6}{l_\mathrm{r}\left|s-s'\right|} \right)^{1/2} \right. \\
&\left. + \frac{\pi}{2}\kappa^3 \exp\left(\frac{\kappa^2 l_\mathrm{r}\left|s-s'\right|}{6} \right) \mathrm{erfc}\left(\sqrt{\frac{\kappa^2 l_\mathrm{r}\left|s-s'\right|}{6}} \right) \right] \\
\xrightarrow{\text{discretize}} &- l_\mathrm{r}^2 \frac{2\lambda_\mathrm{B} l^2}{9\pi} \sum_{m=2}^{N} \sum_{n=1}^{m-1} \left\langle q_m q_n \right\rangle_q (m-n)^2 \left[\frac{\pi^{1/2}}{4} \left(\frac{6}{x l^2 \left|m-n\right|} \right)^{3/2} \right. \\
&- \frac{\pi^{1/2}}{2}\kappa^2 \left(\frac{6}{x l^2 \left|m-n\right|} \right)^{1/2} \\
&\left. + \frac{\pi}{2}\kappa^3 \exp\left(\frac{(\kappa l)^2 x\left|m-n\right|}{6} \right) \mathrm{erfc}\left(\sqrt{\frac{(\kappa l)^2 x\left|m-n\right|}{6}} \right) \right] \\
= &- l_\mathrm{r}^2 \frac{2\lambda_\mathrm{B}}{9\pi l} \sum_{m=2}^{N} \sum_{n=1}^{m-1} \left\langle q_m q_n \right\rangle_q (m-n)^2 \left[\frac{\pi^{1/2}}{4} \left(\frac{6}{x \left|m-n\right|} \right)^{3/2} \right. \\
&- \frac{\pi^{1/2}}{2}(\kappa l)^2 \left(\frac{6}{x \left|m-n\right|} \right)^{1/2} \\
&\left. + \frac{\pi}{2}(\kappa l)^3 \exp\left(\frac{(\kappa l)^2 x\left|m-n\right|}{6} \right) \mathrm{erfc}\left(\sqrt{\frac{(\kappa l)^2 x\left|m-n\right|}{6}} \right) \right] .
\end{aligned}
\tag{S11}
$$

Following Sawle and Ghosh, we now introduce the matrix elements[S1]

$$
\begin{aligned}
A_{mn}(x, \kappa l) \equiv x^{3/2} &\left[\frac{\pi^{1/2}}{4} \left(\frac{6}{x \left|m-n\right|} \right)^{3/2} \right. \\
&- \frac{\pi^{1/2}}{2}(\kappa l)^2 \left(\frac{6}{x \left|m-n\right|} \right)^{1/2} \\
&\left. + \frac{\pi}{2}(\kappa l)^3 \exp\left(\frac{(\kappa l)^2 x\left|m-n\right|}{6} \right) \mathrm{erfc}\left(\sqrt{\frac{(\kappa l)^2 x\left|m-n\right|}{6}} \right) \right] .
\end{aligned}
\tag{S12}
$$

Then, we get

$$\begin{aligned}
&- l_{\mathrm{r}}^{2} \frac{2\lambda_{\mathrm{B}}}{9\pi l} \sum_{m=2}^{N} \sum_{n=1}^{m-1} \left\langle q_m q_n \right\rangle_q (m-n)^2 \left[\frac{\pi^{1/2}}{4} \left(\frac{6}{x\,|m-n|} \right)^{3/2} \right. \\
&- \frac{\pi^{1/2}}{2} (\kappa l)^2 \left(\frac{6}{x\,|m-n|} \right)^{1/2} \\
&\left. + \frac{\pi}{2} (\kappa l)^3 \exp\left(\frac{(\kappa l)^2 x\,|m-n|}{6} \right) \operatorname{erfc}\left(\sqrt{\frac{(\kappa l)^2 x\,|m-n|}{6}} \right) \right] \\
= &- l_{\mathrm{r}}^{2} \frac{2\lambda_{\mathrm{B}}}{9\pi l} \frac{1}{x^{3/2}} \sum_{m=2}^{N} \sum_{n=1}^{m-1} \left\langle q_m q_n \right\rangle_q (m-n)^2 A_{mn}(x, \kappa l) \\
= &- l_{\mathrm{r}}^{2} \frac{2\lambda_{\mathrm{B}}}{9\pi l} \frac{1}{x^{3/2}} \sum_{m=2}^{N} \sum_{n=1}^{m-1} \left\langle q_m \right\rangle_q \left\langle q_n \right\rangle_q (m-n)^2 A_{mn}(x, \kappa l) .
\end{aligned} \tag{S13}$$

In the last step, we used the fact that the charges at different sites are fluctuating independently in their respective mean-fields ϕ_m, i.e.

$$\left\langle q_m q_n \right\rangle_q = \left\langle q_m \right\rangle_q \left\langle q_n \right\rangle_q \tag{S14}$$

for $m \neq n$. A straightforward calculation shows that the mean charge at site m is given by

$$\left\langle q_m \right\rangle_q = \begin{cases} -1/\left(1+\exp\left(-\phi_m\right)\right), & \text{for acidic residues} \\ 1/\left(1+\exp\left(\phi_m\right)\right), & \text{for basic residues.} \end{cases} \tag{S15}$$

2.1.5 Charge Regulation

Finally, the charge regulation terms, which are linear in $q(s)$, exactly cancel. This is the case because the expectation values can be factorized:

$$\begin{aligned}
&\left\langle (\mathbf{R}(L)-\mathbf{R}(0))^2 \frac{1}{l}\int_0^L \mathrm{d}s\, q(s)\left(\phi(s)-\ln(10)\left[\mathrm{pH}-\mathrm{p}K_{\mathrm{A}}(s)\right]\right)\right\rangle_{\mathrm{r}} \\
&\quad -\left\langle (\mathbf{R}(L)-\mathbf{R}(0))^2\right\rangle_{\mathrm{r}} \left\langle \frac{1}{l}\int_0^L \mathrm{d}s\, q(s)\left(\phi(s)-\ln(10)\left[\mathrm{pH}-\mathrm{p}K_{\mathrm{A}}(s)\right]\right)\right\rangle_{\mathrm{r}} \\
&= \left\langle (\mathbf{R}(L)-\mathbf{R}(0))^2\right\rangle_{\mathbf{R}} \left\langle \frac{1}{l}\int_0^L \mathrm{d}s\, q(s)\left(\phi(s)-\ln(10)\left[\mathrm{pH}-\mathrm{p}K_{\mathrm{A}}(s)\right]\right)\right\rangle_{q} \\
&\quad -\left\langle (\mathbf{R}(L)-\mathbf{R}(0))^2\right\rangle_{\mathbf{R}} \left\langle \frac{1}{l}\int_0^L \mathrm{d}s\, q(s)\left(\phi(s)-\ln(10)\left[\mathrm{pH}-\mathrm{p}K_{\mathrm{A}}(s)\right]\right)\right\rangle_{q} \\
&= 0.
\end{aligned} \tag{S16}$$

2.1.6 Combining Everything

Combining the non-vanishing terms (Equations (S7), (S9) and (S13)) results in the following nonlinear equation:

$$\begin{aligned}
&N\left(\frac{1}{x}-1\right)+\left(\frac{3}{2\pi}\right)^{3/2}\frac{1}{x^{5/2}}\sum_{m=2}^{N}\sum_{n=1}^{m-1}\frac{\omega_{mn}}{(m-n)^{1/2}} \\
&+\frac{2\lambda_{\mathrm{B}}}{9\pi l}\frac{1}{x^{3/2}}\sum_{m=2}^{N}\sum_{n=1}^{m-1}\left\langle q_m\right\rangle_q \left\langle q_n\right\rangle_q (m-n)^2 A_{mn}\left(x,\kappa l\right)=0.
\end{aligned} \tag{S17}$$

Here, the mean charge at the site m is given by Equation S15.

2.2 Variational Calculation of Charge State

2.2.1 Preliminaries

We now proceed to derive an explicit expression for Equation 9. $\langle q(u)\left(H_{\mathrm{r}}-H_{\mathrm{t}}\right)\rangle_{\mathrm{r}}$ can be divided into different contributions:

$$\begin{aligned}
&\langle q(u)\left(H_{\mathrm{r}}-H_{\mathrm{t}}\right)\rangle_{\mathrm{r}} \\
=&\left\langle q(u)\frac{3}{2}\left(\frac{1}{l_{\mathrm{r}}}-\frac{1}{l}\right)\int_0^L \mathrm{d}s\left(\frac{\mathrm{d}\mathbf{R}(s)}{\mathrm{d}s}\right)^2\right\rangle_{\mathrm{r}} \\
&-\left\langle q(u)l\int_0^L \mathrm{d}s\int_0^s \mathrm{d}s'\omega(s,s')\delta\left(\mathbf{R}(s)-\mathbf{R}(s')\right)\right\rangle_{\mathrm{r}} \\
&-\left\langle q(u)\frac{\lambda_{\mathrm{B}}}{l^2}\int_0^L \mathrm{d}s\int_0^s \mathrm{d}s' q(s)q(s')\frac{\exp\left(-\kappa\left|\mathbf{R}(s)-\mathbf{R}(s')\right|\right)}{\left|\mathbf{R}(s)-\mathbf{R}(s')\right|}\right\rangle_{\mathrm{r}} \\
&+\left\langle q(u)\frac{1}{l}\int_0^L \mathrm{d}s\, q(s)\left(\phi(s)-\ln(10)\left[\mathrm{pH}-\mathrm{p}K_{\mathrm{A}}(s)\right]\right)\right\rangle_{\mathrm{r}}.
\end{aligned} \tag{S18}$$

2.2.2 Harmonic Bonds

The terms involving the bonded interactions exactly cancel, because the trial Hamiltonian does not couple conformations and ionization states:

$$\begin{aligned}
&\left\langle q(u)\frac{3}{2}\left(\frac{1}{l_{\mathrm{r}}}-\frac{1}{l}\right)\int_0^L \mathrm{d}s\left(\frac{\mathrm{d}\mathbf{R}(s)}{\mathrm{d}s}\right)^2\right\rangle_{\mathrm{r}} \\
&-\langle q(u)\rangle_{\mathrm{r}}\left\langle \frac{3}{2}\left(\frac{1}{l_{\mathrm{r}}}-\frac{1}{l}\right)\int_0^L \mathrm{d}s\left(\frac{\mathrm{d}\mathbf{R}(s)}{\mathrm{d}s}\right)^2\right\rangle_{\mathrm{r}} \\
=&\langle q(u)\rangle_q\left\langle \frac{3}{2}\left(\frac{1}{l_{\mathrm{r}}}-\frac{1}{l}\right)\int_0^L \mathrm{d}s\left(\frac{\mathrm{d}\mathbf{R}(s)}{\mathrm{d}s}\right)^2\right\rangle_{\mathbf{R}} \\
&-\langle q(u)\rangle_q\left\langle \frac{3}{2}\left(\frac{1}{l_{\mathrm{r}}}-\frac{1}{l}\right)\int_0^L \mathrm{d}s\left(\frac{\mathrm{d}\mathbf{R}(s)}{\mathrm{d}s}\right)^2\right\rangle_{\mathbf{R}} \\
=&0.
\end{aligned} \tag{S19}$$

2.2.3 Excluded Volume Interactions

Likewise, the terms involving the excluded volume interactions also exactly cancel:

$$\begin{aligned}
&\left\langle q(u) l \int_0^L \mathrm{d}s \int_0^s \mathrm{d}s' \omega(s,s') \delta\left(\mathbf{R}(s) - \mathbf{R}(s')\right) \right\rangle_{\mathrm{r}} \\
&\quad - \left\langle q(u) \right\rangle_{\mathrm{r}} \left\langle l \int_0^L \mathrm{d}s \int_0^s \mathrm{d}s' \omega(s,s') \delta\left(\mathbf{R}(s) - \mathbf{R}(s')\right) \right\rangle_{\mathrm{r}} \\
=& \left\langle q(u) \right\rangle_q \left\langle l \int_0^L \mathrm{d}s \int_0^s \mathrm{d}s' \omega(s,s') \delta\left(\mathbf{R}(s) - \mathbf{R}(s')\right) \right\rangle_{\mathbf{R}} \\
&\quad - \left\langle q(u) \right\rangle_q \left\langle l \int_0^L \mathrm{d}s \int_0^s \mathrm{d}s' \omega(s,s') \delta\left(\mathbf{R}(s) - \mathbf{R}(s')\right) \right\rangle_{\mathbf{R}} \\
=& 0.
\end{aligned} \tag{S20}$$

2.2.4 Electrostatic Interactions

The terms involving the Debye-Hückel potential can be written as follows.

$$\begin{aligned}
&\left\langle q(u) \frac{\lambda_{\mathrm{B}}}{l^2} \int_0^L \mathrm{d}s \int_0^s \mathrm{d}s' q(s) q(s') \frac{\exp\left(-\kappa \left|\mathbf{R}(s) - \mathbf{R}(s')\right|\right)}{\left|\mathbf{R}(s) - \mathbf{R}(s')\right|} \right\rangle_{\mathrm{r}} \\
&- \left\langle q(u) \right\rangle_{\mathrm{r}} \left\langle \frac{\lambda_{\mathrm{B}}}{l^2} \int_0^L \mathrm{d}s \int_0^s \mathrm{d}s' q(s) q(s') \frac{\exp\left(-\kappa \left|\mathbf{R}(s) - \mathbf{R}(s')\right|\right)}{\left|\mathbf{R}(s) - \mathbf{R}(s')\right|} \right\rangle_{\mathrm{r}} \\
=& \left\langle \frac{\lambda_{\mathrm{B}}}{l^2} \int_0^L \mathrm{d}s \int_0^s \mathrm{d}s' \left(\left\langle q(u) q(s) q(s') \right\rangle_q - \left\langle q(u) \right\rangle_q \left\langle q(s) q(s') \right\rangle_q \right) \times \right. \\
&\left. \times \frac{\exp\left(-\kappa \left|\mathbf{R}(s) - \mathbf{R}(s')\right|\right)}{\left|\mathbf{R}(s) - \mathbf{R}(s')\right|} \right\rangle_{\mathbf{R}}.
\end{aligned} \tag{S21}$$

We now use the Fourier representation of the Debye-Hückel potential,[S1]

$$\frac{\exp\left(-\kappa r\right)}{r} = \int \frac{\mathrm{d}^3 k}{2\pi^2} \frac{\exp\left(i \mathbf{k} \cdot \mathbf{r}\right)}{k^2 + \kappa^2}, \tag{S22}$$

to further simplify the expression:

$$
\begin{aligned}
&\left\langle \frac{\lambda_{\mathrm{B}}}{l^2} \int_0^L \mathrm{d}s \int_0^s \mathrm{d}s' \left(\left\langle q(u)q(s)q(s') \right\rangle_q - \left\langle q(u) \right\rangle_q \left\langle q(s)q(s') \right\rangle_q \right) \times \right. \\
&\left. \times \frac{\exp\left(-\kappa \left|\mathbf{R}(s) - \mathbf{R}(s')\right|\right)}{\left|\mathbf{R}(s) - \mathbf{R}(s')\right|} \right\rangle_{\mathbf{R}} \\
=&\left\langle \frac{\lambda_{\mathrm{B}}}{l^2} \int_0^L \mathrm{d}s \int_0^s \mathrm{d}s' \left(\left\langle q(u)q(s)q(s') \right\rangle_q - \left\langle q(u) \right\rangle_q \left\langle q(s)q(s') \right\rangle_q \right) \times \right. \\
&\left. \times \int \frac{\mathrm{d}^3 k}{2\pi^2} \frac{\exp\left(i\mathbf{k} \cdot \left(\mathbf{R}(s) - \mathbf{R}(s')\right)\right)}{k^2 + \kappa^2} \right\rangle_{\mathbf{R}} \\
=&\frac{\lambda_{\mathrm{B}}}{l^2} \int_0^L \mathrm{d}s \int_0^s \mathrm{d}s' \left(\left\langle q(u)q(s)q(s') \right\rangle_q - \left\langle q(u) \right\rangle_q \left\langle q(s)q(s') \right\rangle_q \right) \times \\
&\times \int \frac{\mathrm{d}^3 k}{2\pi^2} \frac{\left\langle \exp\left(i\mathbf{k} \cdot \left(\mathbf{R}(s) - \mathbf{R}(s')\right)\right) \right\rangle_{\mathbf{R}}}{k^2 + \kappa^2} .
\end{aligned}
\tag{S23}
$$

Using the following relation for a Gaussian chain[S2]

$$
\left\langle \exp\left(i\mathbf{k} \cdot \left(\mathbf{R}(s) - \mathbf{R}(s')\right)\right) \right\rangle_{\mathbf{R}} = \exp\left(-\frac{k^2 l_{\mathrm{r}} \left|s - s'\right|}{6} \right)
\tag{S24}
$$

we get

$$
\begin{aligned}
&\frac{\lambda_{\mathrm{B}}}{l^2} \int_0^L \mathrm{d}s \int_0^s \mathrm{d}s' \left(\left\langle q(u)q(s)q(s') \right\rangle_q - \left\langle q(u) \right\rangle_q \left\langle q(s)q(s') \right\rangle_q \right) \times \\
&\times \int \frac{\mathrm{d}^3 k}{2\pi^2} \frac{\left\langle \exp\left(i\mathbf{k} \cdot \left(\mathbf{R}(s) - \mathbf{R}(s')\right)\right) \right\rangle_{\mathbf{R}}}{k^2 + \kappa^2} \\
=&\frac{\lambda_{\mathrm{B}}}{l^2} \int_0^L \mathrm{d}s \int_0^s \mathrm{d}s' \left(\left\langle q(u)q(s)q(s') \right\rangle_q - \left\langle q(u) \right\rangle_q \left\langle q(s)q(s') \right\rangle_q \right) \times \\
&\times \int \frac{\mathrm{d}^3 k}{2\pi^2} \frac{\exp\left(-\frac{k^2 l_{\mathrm{r}} |s - s'|}{6} \right)}{k^2 + \kappa^2} .
\end{aligned}
\tag{S25}
$$

The k-space integral can be evaluated in closed form using the identity[S3]

$$
\int_0^\infty \frac{\mathrm{d}k\, k^2}{k^2 + \kappa^2} \exp\left(-k^2 y\right) = \frac{\pi^{1/2}}{2} \frac{1}{y^{1/2}} - \frac{\pi}{2} \kappa \exp\left(\kappa^2 y\right) \operatorname{erfc}\left(\sqrt{\kappa^2 y}\right) ,
\tag{S26}
$$

resulting in

$$\begin{aligned}
&\frac{\lambda_{\mathrm{B}}}{l^2}\int_0^L \mathrm{d}s\int_0^s \mathrm{d}s'\left(\left\langle q(u)q(s)q(s')\right\rangle_q - \left\langle q(u)\right\rangle_q\left\langle q(s)q(s')\right\rangle_q\right)\times\\
&\times\int\frac{\mathrm{d}^3k}{2\pi^2}\frac{\exp\left(-\frac{k^2 l_{\mathrm{r}}|s-s'|}{6}\right)}{k^2+\kappa^2}\\
=&\frac{2\lambda_{\mathrm{B}}}{\pi l^2}\int_0^L \mathrm{d}s\int_0^s \mathrm{d}s'\left(\left\langle q(u)q(s)q(s')\right\rangle_q - \left\langle q(u)\right\rangle_q\left\langle q(s)q(s')\right\rangle_q\right)\times\\
&\times\left[\frac{\pi^{1/2}}{2}\left(\frac{6}{l_{\mathrm{r}}\left|s-s'\right|}\right)^{1/2}\right.\\
&\left.-\frac{\pi}{2}\kappa\exp\left(\frac{\kappa^2 l_{\mathrm{r}}\left|s-s'\right|}{6}\right)\operatorname{erfc}\left(\sqrt{\frac{\kappa^2 l_{\mathrm{r}}\left|s-s'\right|}{6}}\right)\right].
\end{aligned}\tag{S27}$$

Discretizing the chain, introducing the abbreviation $x = l_{\mathrm{r}}/l$ and introducing the indices $m = s/l$, $n = s'/l$, $i = u/l$, we arrive at

$$\begin{aligned}
&\frac{2\lambda_{\mathrm{B}}}{\pi l^2}\int_0^L \mathrm{d}s\int_0^s \mathrm{d}s'\left(\left\langle q(u)q(s)q(s')\right\rangle_q - \left\langle q(u)\right\rangle_q\left\langle q(s)q(s')\right\rangle_q\right)\times\\
&\times\left[\frac{\pi^{1/2}}{2}\left(\frac{6}{l_{\mathrm{r}}\left|s-s'\right|}\right)^{1/2}\right.\\
&\left.-\frac{\pi}{2}\kappa\exp\left(\frac{\kappa^2 l_{\mathrm{r}}\left|s-s'\right|}{6}\right)\operatorname{erfc}\left(\sqrt{\frac{\kappa^2 l_{\mathrm{r}}\left|s-s'\right|}{6}}\right)\right]\\
\xrightarrow{\text{discretize}}&\frac{2\lambda_{\mathrm{B}}}{\pi l}\sum_{m=2}^{N}\sum_{n=1}^{m-1}\left(\left\langle q_i q_m q_n\right\rangle_q - \left\langle q_i\right\rangle_q\left\langle q_m q_n\right\rangle_q\right)\left[\frac{\pi^{1/2}}{2}\left(\frac{6}{x\left|m-n\right|}\right)^{1/2}\right.\\
&\left.-\frac{\pi}{2}\kappa l\exp\left(\frac{(\kappa l)^2 x\left|m-n\right|}{6}\right)\operatorname{erfc}\left(\sqrt{\frac{(\kappa l)^2 x\left|m-n\right|}{6}}\right)\right].
\end{aligned}\tag{S28}$$

As an abbreviation, we now define the matrix elements

$$\begin{aligned}
J_{mn}(x,\kappa l) \equiv&\frac{\pi^{1/2}}{2}\left(\frac{6}{x\left|m-n\right|}\right)^{1/2}\\
&-\frac{\pi}{2}\kappa l\exp\left(\frac{(\kappa l)^2 x\left|m-n\right|}{6}\right)\operatorname{erfc}\left(\sqrt{\frac{(\kappa l)^2 x\left|m-n\right|}{6}}\right).
\end{aligned}\tag{S29}$$

Then, we get

$$\begin{aligned}
&\frac{2\lambda_{\mathrm{B}}}{\pi l}\sum_{m=2}^{N}\sum_{n=1}^{m-1}\left(\left\langle q_i q_m q_n\right\rangle_q-\left\langle q_i\right\rangle_q\left\langle q_m q_n\right\rangle_q\right)\left[\frac{\pi^{1/2}}{2}\left(\frac{6}{x\left|m-n\right|}\right)^{1/2}\right.\\
&\left.-\frac{\pi}{2}\kappa l\exp\left(\frac{\left(\kappa l\right)^2 x\left|m-n\right|}{6}\right)\operatorname{erfc}\left(\sqrt{\frac{\left(\kappa l\right)^2 x\left|m-n\right|}{6}}\right)\right]\\
=&\frac{2\lambda_{\mathrm{B}}}{\pi l}\sum_{m=2}^{N}\sum_{n=1}^{m-1}\left(\left\langle q_i q_m q_n\right\rangle_q-\left\langle q_i\right\rangle_q\left\langle q_m q_n\right\rangle_q\right)J_{mn}\left(x,\kappa l\right).
\end{aligned} \tag{S30}$$

To simplify this expression further, we use the fact that the charges at different sites are fluctuating independently in their respective mean-fields, i.e. we have

$$\left\langle q_i q_m q_n\right\rangle_q-\left\langle q_i\right\rangle_q\left\langle q_m q_n\right\rangle_q=0 \tag{S31}$$

if $i \neq m$ and $i \neq n$. This means that we can write the 3-point correlation function in the following form (note that we always have $m \neq n$, because there are no self-interactions in the double sum):

$$\begin{aligned}
&\left\langle q_i q_m q_n\right\rangle_q-\left\langle q_i\right\rangle_q\left\langle q_m q_n\right\rangle_q\\
=&\delta_{i,m}\left(\left\langle q_i q_m q_n\right\rangle_q-\left\langle q_i\right\rangle_q\left\langle q_m q_n\right\rangle_q\right)+\delta_{i,n}\left(\left\langle q_i q_m q_n\right\rangle_q-\left\langle q_i\right\rangle_q\left\langle q_m q_n\right\rangle_q\right)\\
=&\left(\left\langle q_i^2\right\rangle_q-\left\langle q_i\right\rangle_q^2\right)\left(\delta_{i,m}\left\langle q_n\right\rangle_q+\delta_{i,n}\left\langle q_m\right\rangle_q\right).
\end{aligned} \tag{S32}$$

Inserting this expression results in

$$\begin{aligned}
&\frac{2\lambda_{\mathrm{B}}}{\pi l}\sum_{m=2}^{N}\sum_{n=1}^{m-1}\left(\left\langle q_i q_m q_n\right\rangle_q-\left\langle q_i\right\rangle_q\left\langle q_m q_n\right\rangle_q\right)J_{mn}\left(x,\kappa l\right)\\
=&\left(\left\langle q_i^2\right\rangle_q-\left\langle q_i\right\rangle_q^2\right)\frac{2\lambda_{\mathrm{B}}}{\pi l}\sum_{m=2}^{N}\sum_{n=1}^{m-1}\left(\delta_{i,m}\left\langle q_n\right\rangle_q+\delta_{i,n}\left\langle q_m\right\rangle_q\right)J_{mn}\left(x,\kappa l\right)\\
=&\left(\left\langle q_i^2\right\rangle_q-\left\langle q_i\right\rangle_q^2\right)\frac{2\lambda_{\mathrm{B}}}{\pi l}\sum_{\substack{m=1\\ m\neq i}}^{N}\left\langle q_m\right\rangle_q J_{mi}\left(x,\kappa l\right).
\end{aligned} \tag{S33}$$

2.2.5 Charge Regulation

Finally, the charge regulation terms do not depend on the chain conformation and thus become

$$\begin{aligned}
&\left\langle q(u)\frac{1}{l}\int_0^L \mathrm{d}s\, q(s)\left(\phi(s)-\ln(10)\left[\mathrm{pH}-\mathrm{p}K_\mathrm{A}(s)\right]\right)\right\rangle_\mathrm{r} \\
&\quad - \langle q(u)\rangle_\mathrm{r}\left\langle \frac{1}{l}\int_0^L \mathrm{d}s\, q(s)\left(\phi(s)-\ln(10)\left[\mathrm{pH}-\mathrm{p}K_\mathrm{A}(s)\right]\right)\right\rangle_\mathrm{r} \\
&= \left\langle q(u)\frac{1}{l}\int_0^L \mathrm{d}s\, q(s)\left(\phi(s)-\ln(10)\left[\mathrm{pH}-\mathrm{p}K_\mathrm{A}(s)\right]\right)\right\rangle_q \\
&\quad - \langle q(u)\rangle_\mathrm{r}\left\langle \frac{1}{l}\int_0^L \mathrm{d}s\, q(s)\left(\phi(s)-\ln(10)\left[\mathrm{pH}-\mathrm{p}K_\mathrm{A}(s)\right]\right)\right\rangle_q \\
&= \frac{1}{l}\int_0^L \mathrm{d}s\, \left(\langle q(u)q(s)\rangle_q - \langle q(u)\rangle_q \langle q(s)\rangle_q\right)\left(\phi(s)-\ln(10)\left[\mathrm{pH}-\mathrm{p}K_\mathrm{A}(s)\right]\right).
\end{aligned} \tag{S34}$$

Again discretizing the chain and introducing the indices $m = s/l$, $i = u/l$, we obtain

$$\begin{aligned}
&\frac{1}{l}\int_0^L \mathrm{d}s\, \left(\langle q(u)q(s)\rangle_q - \langle q(u)\rangle_q \langle q(s)\rangle_q\right)\left(\phi(s)-\ln(10)\left[\mathrm{pH}-\mathrm{p}K_\mathrm{A}(s)\right]\right) \\
&\xrightarrow{\text{discretize}} \sum_{m=1}^{N}\left(\langle q_i q_m\rangle_q - \langle q_i\rangle_q\langle q_m\rangle_q\right)\left(\phi_m - \ln(10)\left(\mathrm{pH}-\mathrm{p}K_{\mathrm{A},m}\right)\right).
\end{aligned} \tag{S35}$$

Using the fact that the charges are fluctuating independently in their respective mean-fields, we can write the two-point correlation function as

$$\langle q_i q_m\rangle_q - \langle q_i\rangle_q\langle q_m\rangle_q = \delta_{i,m}\left(\left\langle q_i^2\right\rangle_q - \langle q_i\rangle_q^2\right). \tag{S36}$$

Inserting this expression into the sum, we arrive at

$$\begin{aligned}
&\sum_{m=1}^{N}\left(\langle q_i q_m\rangle_q - \langle q_i\rangle_q\langle q_m\rangle_q\right)\left(\phi_m - \ln(10)\left(\mathrm{pH}-\mathrm{p}K_{\mathrm{A},m}\right)\right) \\
&= \sum_{m=1}^{N}\delta_{i,m}\left(\left\langle q_i^2\right\rangle_q - \langle q_i\rangle_q^2\right)\left(\phi_m - \ln(10)\left(\mathrm{pH}-\mathrm{p}K_{\mathrm{A},m}\right)\right) \\
&= \left(\left\langle q_i^2\right\rangle_q - \langle q_i\rangle_q^2\right)\left(\phi_i - \ln(10)\left(\mathrm{pH}-\mathrm{p}K_{\mathrm{A},i}\right)\right).
\end{aligned} \tag{S37}$$

2.2.6 Combining Everything

Combining Equation S33 and Equation S37, we end up with

$$\begin{aligned}&\left(\left\langle q_i^2\right\rangle_q - \left\langle q_i\right\rangle_q^2\right)\left(\phi_i - \ln(10)\left(\mathrm{pH} - \mathrm{p}K_{\mathrm{A},i}\right)\right)\\&-\left(\left\langle q_i^2\right\rangle_q - \left\langle q_i\right\rangle_q^2\right)\frac{2\lambda_\mathrm{B}}{\pi l}\sum_{\substack{m=1\\m\neq i}}^{N}\left\langle q_m\right\rangle_q J_{mi}\left(x,\kappa l\right) = 0.\end{aligned}\tag{S38}$$

Because $\left(\left\langle q_i^2\right\rangle_q - \left\langle q_i\right\rangle_q^2\right) > 0$, this equation reduces to

$$\phi_i = \ln(10)\left(\mathrm{pH} - \mathrm{p}K_{\mathrm{A},i}\right) + \frac{2\lambda_\mathrm{B}}{\pi l}\sum_{\substack{m=1\\m\neq i}}^{N}\left\langle q_m\right\rangle_q J_{mi}\left(x,\kappa l\right).\tag{S39}$$

References

(S1) Sawle, L.; Ghosh, K. A theoretical method to compute sequence dependent configurational properties in charged polymers and proteins. *The Journal of Chemical Physics* **2015**, *143*, 085101.

(S2) Doi, M.; Edwards, S. *The Theory of Polymer Dynamics*, 1st ed.; Oxford University Press: Oxford, 1988.

(S3) Meurer, A.; Smith, C. P.; Paprocki, M.; Čertík, O.; Kirpichev, S. B.; Rocklin, M.; Kumar, A.; Ivanov, S.; Moore, J. K.; Singh, S.; Rathnayake, T.; Vig, S.; Granger, B. E.; Muller, R. P.; Bonazzi, F.; Gupta, H.; Vats, S.; Johansson, F.; Pedregosa, F.; Curry, M. J.; Terrel, A. R.; Roučka, v.; Saboo, A.; Fernando, I.; Kulal, S.; Cimrman, R.; Scopatz, A. SymPy: symbolic computing in Python. *PeerJ Computer Science* **2017**, *3*, e103.
